# Neurodevelopmental outcomes of neonatal posthemorrhagic hydrocephalus and psychological effects on the parents

**DOI:** 10.1007/s00381-023-05935-y

**Published:** 2023-04-20

**Authors:** Yuxin Wu, Ping Liang, Lusheng Li, Yudong Zhou, Difei Wang, Xuan Zhai

**Affiliations:** Department of Neurosurgery, Ministry of Education Key Laboratory of Child Development and Disorders, National Clinical Research Center for Child Health and Disorders, China International Science and Technology Cooperation Base of Child Development and Critical Disorders, Chongqing Key Laboratory of Pediatrics, Children’s Hospital of Chongqing Medical University, No.136, Zhongshan 2nd Road, Yuzhong District, Chongqing, 400010 China

**Keywords:** Intraventricular haemorrhage, Posthemorrhagic hydrocephalus, Outcomes, Anxiety, Depression

## Abstract

**Background:**

Neonatal posthemorrhagic hydrocephalus remains a common complication in preterm infants, with high rates of mortality and morbidity, placing parents at high risk of anxiety and depression. We sought to investigate the neurodevelopmental outcomes of infants with posthemorrhagic hydrocephalus who underwent surgery and the psychological effect on their parents.

**Methods:**

We retrospectively analysed all infants with posthemorrhagic hydrocephalus born between 2014 and 2020 in the Children’s Hospital of Chongqing Medical University, China. The neurodevelopmental outcomes of 28 patients were evaluated by the Pediatric Stroke Outcome Measure score, and the psychological states of the parents of survivors were assessed by the Hospital Anxiety and Depression Scale.

**Results:**

The families of the 28 patients were followed up for a median duration of 3 years; 6 (21.4%) patients died within 6 months after discharge, 12 (42.9%) patients had moderate to severe dysfunction, and only 10 (35.7%) patients had good outcomes. Regarding the 22 parents of the survivors, 5 (22.7%) and 4 (18.2%) had borderline anxiety and depression symptoms, respectively. Two (9.1%) caregivers had exact anxiety and depression symptoms. Leukomalacia after intraventricular haemorrhage was associated with adverse neurological outcomes. The infants' histories of epileptic seizures during the neonatal period were associated with the anxiety of their parents.

**Conclusion:**

The overall outcome of posthemorrhagic hydrocephalus patients is unsatisfactory, and children with leukomalacia after haemorrhage tend to have poor outcomes. A history of epileptic seizures during the course of the disease may exacerbate the anxiety of the caregivers.

## Introduction

Intraventricular haemorrhage (IVH) is a common but devastating complication for preterm infants, resulting in neonatal mortality and morbidity [1, 2]. The inherent fragility of the vascularized germinal matrix and disturbance of cerebral blood flow contribute to IVH [3]. Blood deposits in the ventricles impede cerebrospinal fluid (CSF) flow and trigger inflammation of the ventricular canal. This causes fluctuations in cerebral blood flow and the ability of oxygen to reach the brain, which finally leads to hydrocephalus [4]. Hydrocephalus is the most common complication of IVH and is more likely to develop in higher grades, affecting 25% and 28% of individuals with grades III and IV, respectively [5, 6].

Posthemorrhagic hydrocephalus (PHH) may lead to a series of neurodevelopmental disorders, such as motor dysfunction, intellectual disability, behavioural problems, memory and executive deficits, emotional problems such as anxiety and depression, attention deficit hyperactivity disorder, and visual impairment. Depending on the degree of brain developmental impairment, motor dysfunction may range from mild motor dysfunction and fine motor incoordination to cerebral palsy [4, 7]. With progress in the management of IVH/PHH, our treatment goals should change from draining cerebrospinal fluid (CSF) or decreasing intracranial pressure, thus saving lives, to improving cognitive function, neurodevelopmental outcomes and quality of life for neonates with IVH. However, most previous studies focused on surgical complications such as CSF infection, the shunt conversion rate, catheter blockage and catheter revision while ignoring neurodevelopmental outcomes, or the evaluation of neurological outcome was not sufficiently comprehensive and only included motor and language functions [8–14]. Statistics related to IVH from the Nationwide Inpatient Sample and the Kids' Inpatient Database showed that thanks to progress in perinatal and neonatal care, the number of preterm infants is decreasing, but the number of newborns hospitalized for IVH/PHH and the length of hospitalization for IVH patients have increased [5].  This suggests that IVH/PHH has become a serious public health problem, inducing not only high rates of neurodevelopmental disorders in children but also increased hospitalization, cost and prolonged hospitalization, which together constitute a source of parental stress.

The aim of our study was to investigate the neurodevelopmental outcomes of infants with posthemorrhagic hydrocephalus who underwent surgery and the psychological effect on their parents.

## Methods

### Baseline demographic data

We retrospectively reviewed 31 PHH patients in the Neurosurgery Ward at the Children’s Hospital of Chongqing Medical University between May 2014 and May 2020 who met the following inclusion criteria: computed tomography (CT) and craniocerebral ultrasound data, previous CSF drainage treatment, and a follow-up interval of at least 1 year. PHH was diagnosed by a neurosurgeon and radiologist according to cranial ultrasound and CT scans during hospitalization. Computed tomography angiography (CTA) was performed if underlying vascular lesions were suspected. Two patients were excluded due to missing contact information, and one patient was excluded because of loss to follow-up. We extracted baseline information, including demographic data, presentations, complications and the type of drainage device, from the clinical records. Newborns with a birth weight of less than 2500 g were defined as having a low birth weight. IVH was graded by the modified Papile classification, [15] and the haemorrhage site was divided into the supratentorial ventricle, infratentorial ventricle and whole ventricle.

### Outcome definitions

The Pediatric Stroke Outcome Measure (PSOM), a standardized score that comes in two versions, one for children aged 0 to 2 years and another for children aged older than 2 years, was used to assess the neurodevelopmental outcomes of patients. The PSOM score is scored from 0 to 2 in 5 domains (right and left sensorimotor, expressive and receptive language, cognitive/behavioural), and the outcome was classified into two categories according to the PSOM score: a good outcome (normal or mild deficits) and a poor outcome (moderate or severe deficits or death) [16]. The Hospital Anxiety and Depression Scale (HADS) was used to assess the psychological states of the parents of surviving children (n = 22), reflecting the impact of PHH on families. The Hospital Anxiety and Depression Scale (HADS) [17] is a screening table invented in 1983 for anxiety and depression that has been widely adopted in general hospitals. The HADS consists of 14 items and two subscales (7 items each) that correspond to anxiety and depression, and each item contains 4 options and is scored from 0 to 3. Outcomes were defined as normal (total score 0–7), borderline anxiety or depressive symptoms (total score 8–10), and definitive symptoms of anxiety or depression (total score > 11). The HADS has been used to characterize the impact of children’s disease on parents’ psychological states [18, 19].

### Statistical analyses

Data were analysed using IBM SPSS Statistics 22. Data are expressed as the median or arithmetic mean ± standard deviation, with the range (minimum–maximum) noted in parentheses. The associations between neurological outcomes and dichotomous variables were assessed by Fisher's exact test, and the Mann‒Whitney U test was used to assess the associations between the HADS score and dichotomous variables. A two-tailed p value was used, and p values < 0.05 were considered statistically significant.

### Ethical statement

This study was approved by the Ethics Committee of the Children’s Hospital of Chongqing Medical University. All data were anonymized.

## Results

### Demographic data

Thirty-one newborns met our inclusion criteria (Table 1), and 3 patients were excluded. Twenty-three (82.1%) patients were male, and 5 (17.9%) patients were female. All patients were transferred from other hospitals after birth. Gestational age (GA) ranged from 30 to 41 weeks (mean 37 ± 3 weeks), and 22 infants (78.6%) were delivered at term according to the medical records. The mean birth weight (BW) was 2986 ± 577 g (range 1780–33,850 g). Among these 28 patients, 13 (46.4%) presented with epilepsy, 13 (46.4%) presented with poor feeding, 4 (14.3%) presented with vomiting, 10 (35.7%) had a bulging fontanel and 4 (14.3%) presented with apnoea. According to the cranial ultrasound, all patients had high-grade IVH: 16 (57.1%) patients had grade III IVH, and 12 (42.9%) patients had grade IV IVH. IVH was isolated in the supratentorial ventricle in 25 (89.3%) patients, the whole ventricle was involved in 3 (10.7%) patients, and no isolated infratentorial ventricular haemorrhage was present. Leukomalacia after haemorrhage was found in 8 (28.6%) patients by cranial ultrasound and magnetic resonance imaging (MRI).

**Table 1:** Baseline characteristics and neurological outcomes in neonatal patients with PHH

Variables (n, %)	Favourable outcome N = 10	Poor outcome N = 18	P value
**Demographic features**			
Male gender	8 (80.0)	15 (83.3)	1.00
Preterm birth	1 (10.0)	5 (27.8)	0.38
Low birth weight	1 (10.0)	5 (27.8)	0.38
Primiparity	5 (50.0)	10 (55.6)	1.00
**Initial symptoms**			
Seizure	4 (40.0)	9 (50.0)	0.70
Apnoea	0 (0.0)	4 (22.2)	0.27
Vomiting	2 (20.0)	2 (11.1)	0.60
Bulging fontanel	2 (20.0)	8 (44.4)	0.25
Poor feeding	5 (50.0)	8 (44.4)	1.00
**Complications**			
Encephalomalacia	0 (0.0)	8 (44.4)	0.03*
Anaemia	1 (10.0)	8 (44.4)	0.10
Intracranial infection	1 (10.0)	3 (16.7)	1.00
Hyperbilirubinemia	3 (30.0)	5 (27.8)	1.00
Cerebral hernia	0 (0.0)	1 (5.6)	1.00
**Haemorrhage characteristics**			
Papile grade			0.70
Papile grade III	5 (50.0)	11 (61.1)	
Papile grade IV	5 (50.0)	7 (38.9)	
Bleeding site			0.53
Supratentorial ventricle	10 (10.0)	15 (83.3)	
Whole ventricle	0 (0.0)	3 (16.7)	
**Treatment**			
Interventions			0.74
VAD	7 (70.0)	11 (61.1)	
EVD	0 (0.0)	2 (11.1)	
VAD + EVD	2 (20.0)	3 (16.7)	
VP shunt	1 (10.0)	2 (11.1)	
Secondary surgery	3 (30.0)	3 (18.8)	0.64

### Treatments

Among the 28 PHH newborns, only 2 underwent initial ventriculoperitoneal shunt implantation, and the remaining 26 patients received temporary CSF drainage first. Eighteen (64.3%) received a ventricular assist device (VAD), and 3 (10.7%) were implanted with external ventricular drains (EVDs) as their initial procedure. Five (17.9) patients with severe hydrocephalus required rapid drainage of large amounts of CSF, a VAD was placed on one side of the ventricle, and an EVD was implanted on the other side of the ventricle. Then, the EVD was removed after one week, while the VAD was retained for one month to continue to control intracranial pressure, if necessary. Five of the 18 patients (27.8%) with a VAD and 1 of the 5 patients (20.0%) with a VAD and an EVD received a secondary VP shunt due to progression of the PHH. The initial method of temporary CSF drainage did not significantly impact the VP shunt conversion rate.

### Outcomes

**Fig. 1:**
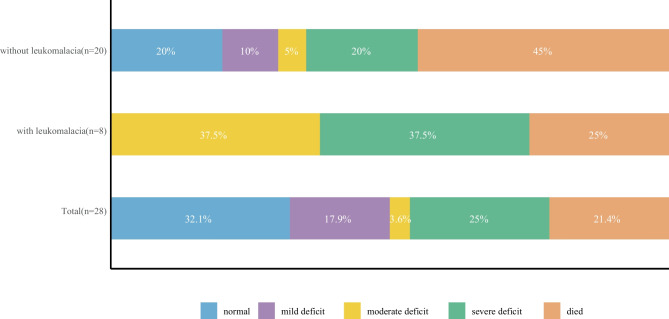
Relationship between leukomalacia and neurological outcomes

**Fig. 2:**
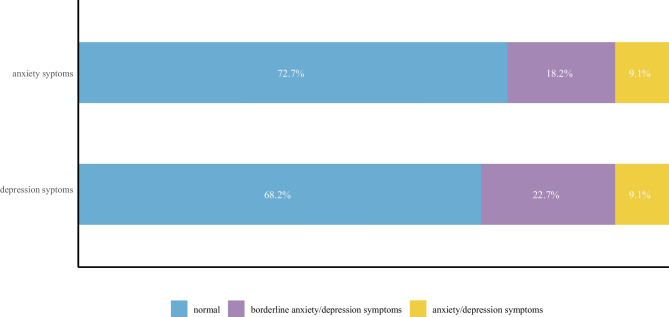
Psychological states of the caregivers of PHH patients

The median follow-up duration was 3 years (22 survivors), with 6 deaths, and all patients had a minimum follow-up of 1 year. Among the six patients who died, 4 patients still had moderate hydrocephalus when they were discharged from the hospital, 1 patient fell into a coma after surgery and was lethargic at discharge, only 1 patient was in good condition at discharge, and all 6 died within 6 months after discharge due to hydrocephalus. The Pediatric Stroke Outcomes Measure scales of these children are shown in Fig. 1. Overall, 10 (35.7%) patients had good neurological outcomes, and 12 (42.8%) had moderate or severe deficits. In our cohort, a risk factor for poor outcomes was leukomalacia (P = 0.03). Among these 22 survivors, 11 (50.0%) patients had motor dysfunction, 4 (18.2%) suffered from language delays, and only 1 patient had a transient seizure at 4 years, with no abnormalities shown on EEG. There was no evidence that secondary surgery influenced the neurological outcomes (P = 0.64). The psychological status of the parents of 22 survivors is shown in Fig. 2. Five in 22 (22.7%) and 4 in 22 (18.2%) had borderline anxiety and depression symptoms, respectively. Two of the 22 (9.1%) caregivers had specific anxiety and depression symptoms, suggesting that their symptoms interfered with daily life.

## Discussion

Intraventricular haemorrhage is frequently accompanied by neurodevelopmental impairments in neonates, both because of the brain damage caused by the haemorrhage and the toxic effects of blood degradation products; 9% of neonates with IVH have hydrocephalus. Elevated intracranial pressure exacerbates damage to the developing neonatal brain [5, 20]. Prior studies have indicated that PHH is associated with cerebral palsy and poorer neurodevelopmental outcomes than in patients without PHH [21, 22]. In our series, death occurred in 6 of the 28 PHH children, and approximately 35% of patients had favourable outcomes. This rate is consistent with the data from Gilard et al., who showed that 31% of children had good outcomes [23]. Encephalomalacia after haemorrhage was a risk factor for poor neurodevelopmental outcomes in our study.

At present, there is no standardized procedure for the treatment of PHH. VP shunts are considered to be the most effective treatment for progressive PHH. However, because of its complications, such as shunt obstruction, shunt infection, and abdominal skin ulcers, VP shunts are dependent on the patients’ physical conditions and thus are not currently regarded as a first-line treatment [9]. In most cases, temporizing neurosurgical procedures (TNPs) for CSF removal are a common treatment, and VP shunts are only performed when TNP cannot control the hydrocephalus [11, 13]. Additionally, it has been reported in the literature that patients requiring VP shunts have a poorer outcome [22]. Therefore, the VP shunt conversion rate is used as an indicator of the therapeutic effect of TNP [9]. In our cohort, 5 out of the 18 (27%) patients who received a VAD and 1 of 5 (20%) patients who underwent VAD + EVD implantation needed VP shunts. Our VP shunt conversion rate is generally in accordance with the rate reported in the literature [23]. Of these six patients who received subsequent VP shunts, half had a favourable outcome. There was no significant difference in the neurological outcomes between patients who received TNP and those who needed VP shunts (P = 0.64). This finding is contrary to that in previous studies. Despite our small sample size, we suspect that the use of the VP shunt conversion rate as an indicator of TNP efficacy is questionable [24, 25].

White matter injury (WMI) was found to be an important pathological alteration in PHH that impedes neonatal brain development. A strong association between IVH and WMI was revealed by an ultrasound screening study on 1064 premature infants; O'Shea et al. proposed that patients with IVH plus WMI have poorer neurodevelopmental outcomes than IVH patients without WMI [26]. Albert M Isaacs et al. applied diffusion basis spectrum imaging to evaluate 95 preterm neonates and found that neonates with PHH had more severe white matter brain damage than those with IVH alone, which explains why neonates with PHH had poorer neurodevelopmental outcomes [27]. WMI is caused by a series of blood-induced pathological reactions, comprising oxidative stress, inflammation, glutamate excitotoxicity, disturbed signalling pathways and remodelling of the extracellular matrix. In the early stage, WMI mainly results from plasma components, including thrombin, immunoglobulins and complement. Cytotoxic haemoglobin and iron released by lysed erythrocytes subsequently exacerbate cerebral injury [2]. An autopsy study of patients who died of IVH indicated that approximately 75% of patients suffered from WMI [28]. Hydrocephalus will develop in approximately 9% of IVH patients, while PHH can induce white matter inflammation and produce further damage to the white matter [29]. Some authors have argued that high-grade IVH, ventricular dilation after bleeding, and the use of VP shunt surgery may not cause poor neurological outcomes but instead increase the risk of WMI, which is the root cause of poor outcomes [9, 26]. Encephalomalacia is a form of white matter injury [26], defined as the softening of the corresponding area caused by liquefaction necrosis of the brain parenchyma and surrounding tissue with or without glial proliferation. In this study, we explored the association between WMI and neurological outcomes of PHH patients. A total of 8 PHH patients were found to have leukomalacia by ultrasound and MRI, two patients died within half a year after surgery, six of the survivors had varying degrees of cerebral palsy, and one of the survivors had delayed speech and WMI associated with poor neurological outcomes (P = 0.03). In our series, we discovered that even with active intervention for hydrocephalus, nearly 40% of the neonates still had unsatisfactory brain developmental outcomes. This finding suggests that the key to preventing injury may not be in controlling intracranial pressure or hydrocephalus but rather in early intervention to remove blood degradation products in the ventricles and reduce encephalomalacia/WMI. This has been recognized in other studies, and new treatments have been devised, such as drainage, irrigation, fibrinolytic therapy (DRIFT), and neuroendoscopic ventricular lavage, to improve long-term outcomes [30]. For neonates with previously detected encephalomalacia, the frequency of follow-up visits should be increased, and rehabilitation should be started early to improve neurological function. Due to the long hospitalization after surgery, high costs, and poor neurodevelopment outcomes, [5] parents are in a state of high mental pressure for a long time. However, previous literature on IVH ignored the influence on the psychological status of parents. In this study, approximately one-third of the caregivers may have had symptoms of anxiety or depression (Table 2), and neonatal epileptic seizures were associated with parents’ anxiety symptoms (P = 0.01). A 2020 prospective cohort study confirmed that 54% of the caregivers of neonates with symptomatic seizures had symptoms of anxiety, and 32% had symptoms of depression [18]. These relationships with neonatal symptomatic seizures may partly be explained by the fact that when we talked to parents about the adverse outcomes of IVH, we mentioned epilepsy, intellectual disability, language delay, etc. Unlike language delay and other obvious sequelae, epilepsy is “unpredictable”; it can happen at any time in the future, similar to a “stealth bomb”. Parents who have witnessed their child have an epileptic seizure are under a greater mental burden, mistakenly believing that their child is more likely to develop epilepsy than other children who do not have a history of seizures. However, is this truly the case? Another interesting result was that in our cohort, acute symptomatic seizures in the neonatal period of IVH patients were not related to remote epilepsy (P = 0.46). Only one patient presented with a seizure again at the age of 4 years, and no similar seizure occurred during the 1-year follow-up. The EEG of the patient was also normal. This epilepsy rate is close to the rate reported by Cole et al. (6%) [31]. Our observations are similar to those of Beslow et al., who previously suggested that having a seizure as the initial presentation of paediatric intracerebral haemorrhage does not increase the risk of remote epilepsy [32]. Therefore, we recommend that when communicating with parents of newborns whose initial symptoms are seizures, it should be explained that this does not mean their child will be at a higher risk of epilepsy in the future.

**Table 2:** Psychological states of the parents of children with PHH

Variables	Anxiety Scales		Depression Scales	
	**Z value**	**P value**	**Z value**	**P value**
**Demographic features**				
Male sex	-0.42	0.68	-0.22	0.83
Preterm	-0.06	0.95	-0.12	0.90
Low birth weight	-0.35	0.73	-0.49	0.62
Primiparity	-0.62	0.52	-0.26	0.80
**Initial symptoms**				
Seizure	-2.60	0.01*	-0.25	0.80
Apnoea	-1.21	0.23	-0.25	0.80
Vomiting	-0.99	0.32	-1.20	0.23
Bulging fontanel	-0.09	0.93	-0.47	0.64
Poor feeding	-1.50	0.13	-1.22	0.22
**Complications**				
Encephalomalacia	-0.81	0.42	-0.47	0.63
Anaemia	-0.09	0.93	-0.45	0.62
Intracranial infection	-0.42	0.68	-0.88	0.38
Hyperbilirubinemia	-0.09	0.93	-0.09	0.93
Cerebral hernia	-1.25	0.21	-0.61	0.55
**Haemorrhage characteristics**				
Papile grade	-0.94	0.35	-1.45	0.15
Bleeding site	-1.25	0.21	-0.61	0.55
**Treatment and Neurodevelopmental Outcomes**				
Secondary surgery	-1.62	0.10	-0.92	0.36
Neurodevelopmental outcomes	-1.20	0.23	-0.84	0.40
**Interviewee information**				
Child age > 3 years old	-0.93	0.36	-0.51	0.61
Caregiver sex	-0.16	0.87	-0.51	0.61

## Conclusion

We conducted a study on 28 neonates with posthemorrhagic hydrocephalus who underwent surgical treatment. Nearly 40% of PHH patients still had significant functional disability after surgery, and leukomalacia after haemorrhage may be a risk factor for adverse neurological outcomes. Therefore, these infants need to be followed up closely. If a functional disability or leukomalacia is found, it is necessary to perform regular rehabilitation to improve their functional status. At the same time, our study suggests that a history of epileptic seizures during the course of the disease is related to the parents’ anxiety symptoms. Regular psychological screening and psychological intervention are necessary for these parents. It is also important to carefully explain the relevant information of IVH during doctor‒patient communication. These measures may help improve the quality of life for the families.

## Limitations

We must admit that our study has limitations. This is a single-centre retrospective study with a small sample size and lacking MRI data for some patients. Despite these limitations, our study still newly proposes that the VP shunt conversion rate may not be a good index for evaluating the TNP effect and provides additional evidence on the association between white matter injury and adverse neurological outcomes. In addition, we have drawn attention to the psychological state of the parents of newborns with intraventricular haemorrhage, which may be beneficial to their later quality of life.

## Data Availability

Data are available on request to the authors.
